# YAP and TAZ play a crucial role in human erythrocyte maturation and enucleation

**DOI:** 10.1186/s13287-022-03166-7

**Published:** 2022-09-08

**Authors:** Nattaya Damkham, Chanchao Lorthongpanich, Phatchanat Klaihmon, Usaneeporn Lueangamornnara, Pakpoom Kheolamai, Kongtana Trakarnsanga, Surapol Issaragrisil

**Affiliations:** 1 Graduate Program in Immunology, Department of Immunology, Faculty of Medicine Siriraj Hospital, Mahidol University, Bangkok, Thailand; 2 Siriraj Center of Excellence for Stem Cell Research, Faculty of Medicine Siriraj Hospital, Mahidol University, 2 Wanglang Road, Siriraj, Bangkoknoi, Bangkok, 10700 Thailand; 3 Division of Hematology, Department of Medicine, Faculty of Medicine Siriraj Hospital, Mahidol University, Bangkok, Thailand; 4 Division of Cell Biology, Department of Pre-Clinical Science, Faculty of Medicine, Thammasat University, Pathum Thani, Thailand; 5 Department of Biochemistry, Faculty of Medicine Siriraj Hospital, Mahidol University, Bangkok, Thailand; 6 Bangkok Hematology Center, Wattanosoth Hospital, BDMS Center of Excellence for Cancer, Bangkok, Thailand

**Keywords:** YAP, TAZ, Hippo pathway, Erythroid differentiation, Hematopoietic stem cells

## Abstract

**Background:**

Yes-associated protein (YAP) and WW domain-containing transcription regulator protein 1 (WWTR1, also known as TAZ) are two key transcription co-activators of the Hippo pathway. Both were originally characterized as organ size and cell proliferation regulators. Later studies demonstrated that the Hippo pathway may play a role in *Drosophila* and mammal hematopoiesis. However, the role of the Hippo pathway in human erythropoiesis has not yet been fully elucidated.

**Methods:**

The role of YAP and TAZ was studied in human erythropoiesis and hematopoietic stem cell (HSC) lineage determination by using mobilized peripheral blood (PB) and cord blood (CB)-derived HSC as a model. HSCs were isolated and cultured in an erythroid differentiation medium for erythroid differentiation and culture in methylcellulose assay for HSC lineage determination study.

**Results:**

YAP and TAZ were barely detectable in human HSCs, but became highly expressed in pro-erythroblasts and erythroblasts. Depletion or knockdown of YAP and/or TAZ did not affect the ability of HSC lineage specification to erythroid lineage in either methylcellulose assay or liquid culture. However, depletion of YAP and TAZ did impair erythroblast terminal differentiation to erythrocytes and their enucleation. Moreover, ectopic expression of YAP and TAZ in pro-erythroblasts did not exert an apparent effect on erythroid differentiation, expansion, or morphology.

**Conclusions:**

This study demonstrated that YAP/TAZ plays important role in erythroid maturation and enucleation but is dispensable for lineage determination of human HSCs.

**Supplementary Information:**

The online version contains supplementary material available at 10.1186/s13287-022-03166-7.

## Key point

YAP and TAZ are required for erythroid maturation and enucleation, but they are dispensable during human HSC lineage allocation to myeloid or erythroid lineages.

**Supplementary Information:**

The online version contains supplementary material available at 10.1186/s13287-022-03166-7.

## Background

There is an increasing worldwide demand for blood transfusion, and donor-blood matching continues to be a problem. Therefore, measures to increase red blood cell production in vitro, including the generation of immortalized adult erythroid progenitor cell lines, are investigated [1]. Erythropoiesis is the process of red blood cell production from hematopoietic stem cells (HSCs) [2]. This differentiation process proceeds under the direction of a complex web of transcription factors.

The Hippo pathway was first discovered in *Drosophila* in 1995 [3, 4] for cell proliferation and organ size regulator [5–8]. YAP and TAZ are two key transcription co-activators of the Hippo signaling pathway in mammals (homologs to Yorkie in *Drosophila*) [9, 10]. Functional redundancy between YAP and TAZ were observed but remains controversial [11–13]. Recently, the novel role of Hippo pathway in *Drosophila* and mammals’ hematopoiesis has been demonstrated. In *Drosophila*, Yorkie and Scalloped (homologs to TEAD in mammals) are required for lineage specification and differentiation of crystal cells, which function like platelet-producing megakaryocytes in mammals [14, 15]. Our research group previously reported that knockdown of large tumor suppressor kinase 1 and 2 (LATS1/2), which are key enzymes of the Hippo pathway, could increase human megakaryocyte biogenesis [16]. Moreover, Yes-associated protein (YAP) plays an essential role in megakaryoblastic cell proliferation, maturation, and platelet production while WW domain-containing transcription regulator protein 1 (WWTR1, also known as TAZ) showed a minor effect [17]. Since megakaryocytes and erythrocytes are developed from a common megakaryocyte-erythroid progenitor, we set forth to study the role of YAP and TAZ, which are downstream effectors of the LATS1/2 kinases and Hippo pathway, in human erythropoiesis.

In this study, we performed loss-and gain-of-function experiments by using genetic manipulation targeting YAP and TAZ using CRISPR/Cas9. In addition, small molecules that mediated YAP/TAZ activity were also used. Lysophosphatidic acid (LPA) was used as a YAP/TAZ activator [18], while Dobutamine hydrochloride (DH) [19] and Verteporfin (VP) were used for inhibiting YAP/TAZ activity [20, 21]. Our result demonstrated that YAP and/or TAZ are required for erythroblast maturation and enucleation, but that they are not necessary for signaling the establishment of erythroid lineage from human HSCs.

## Materials and methods

### Sample collection and CD34^+^ HSC isolation

G-CSF mobilized peripheral blood, and umbilical cord blood were collected from healthy donors under the protocol approved by the Siriraj Institutional Review Board (COA no. Si 711/2018), Faculty of Medicine Siriraj Hospital, Mahidol University, Bangkok, Thailand. Written informed consent was obtained from all donors before blood collection. Mononuclear cells were isolated using Lymphoprep™ density gradient medium (STEMCELL Technologies, Vancouver, Canada). CD34^+^ cells were isolated using a CD34^+^ Microbead Kit (Human) and MS column (Miltenyi Biotec, Bergisch Gladbach, Germany).

### Erythroid differentiation

Purified CD34^+^ HSCs were differentiated in a three-stage erythroid culture system [22]. The basal medium consisted of Iscove’s Modified Dulbecco’s Medium (IMDM, FG0465; Biochrom Ltd, Cambridge, UK) supplemented with 2% heat-inactivated (56 °C, 30 min) fetal bovine serum (FBS; Merck Millipore, Burlington, MA, USA), 3% heat-inactivated human AB serum obtained from human AB blood group donor, 200 μg/ml transferrin (T0665; Sigma-Aldrich, St. Louis, MO, USA), 3 U/ml heparin (Leo Pharma, Ballerup, Denmark), 10 μg/ml insulin (I9278; Sigma-Aldrich), 3 U/ml EPO (Janssen Pharmaceuticals, Beerse, Belgium), 100 U/ml of penicillin (Sigma-Aldrich), and 100 mg/ml streptomycin (Sigma-Aldrich). Stage I (day 0–8), the basal medium was supplemented with 10 ng/ml of Stem Cell Factor (SCF; R&D Systems, Minneapolis, MN, USA) and 1 ng/ml interleukin-3 (IL-3; R&D Systems). Stage II (day 8–11), the basal medium was supplemented with 10 ng/ml SCF and stage III (day 11–20), the basal medium was supplemented with 500 μg/ml transferrin. Purified CD34^+^ HSCs were seeded at a density of 2.5 × 10^4^ cells/ml and maintained at a density of 2–10 × 10^5^ cells/ml by half changed or adding fresh medium every 2–3 days. Cells were incubated at 37 °C in a 5% CO_2_ incubator. Starting at day 7–8, a reddish pellet was observed.

### Lysophosphatidic acid (LPA), dobutamine hydrochloride (DH), and verteporfin (VP) treatment

1-Oleoyl-sn-glycero-3-phosphate (lysophosphatidic acid: LPA, L7260; Sigma-Aldrich), a YAP/TAZ activator, and dobutamine hydrochloride (DH, D0676; Sigma-Aldrich), a YAP/TAZ inhibitor, were added at a final concentration of 10 μM every other day. Verteporfin (VP) (SML0534; Sigma-Aldrich), an inhibitor of YAP/TAZ-TEAD interaction, was added at a final concentration of 1–2 μM every other day in the dark.

### Knockdown of YAP and TAZ in CD34^+^ HSC-derived erythroid cells

For the knockdown of *YAP*, three sgRNA targeted to the *YAP* gene were designed using an online website (CRISPR.mit.edu). Sequences of gRNA-YAP are shown in Additional file 1: Table S1. gRNA-YAP were cloned into pSpCas9(BB)-2A-GFP (PX458-GFP) (#48138; Addgene, Watertown, MA, USA) according to a published protocol [23] and named PX458-GFP_gYAP. sgRNA sequences were validated by Sanger DNA sequencing using U6-Fwd primer (5′-GAGGGCCTATTTCCCATGATTCC-3′) [23]. Nucleofection was performed using an Amaxa 4D Nucleofection System with P3 Primary Solution Kit (Lonza, Basel, Switzerland) according to the manufacturer’s protocols using the DZ100 program and 1 μg plasmid. GFP^+^ cells were sorted using a FACS Aria III Cell Sorter (BD Bioscience) at 24 h after nucleofection and continuously cultured in an erythroid differentiation medium.

*TAZ* (also known as WWTR1) was knocked down using pLenti-Puro_gWWTR1 (GenScript, Piscataway, NJ, USA; gRNA sequence: 5′-TCTCATGTCTGGGGTCATCG-3′) [24]. Lentivirus particles were prepared by transfecting HEK293FT (ATCC, Manassas, VA, USA) with 5 μg pLenti-Puro_gWWTR1, 1 μg pCMV-VSV-G (#12259; Addgene), and 4.17 μg pCMV-dR8.2 (#12263; Addgene) (using Lipofectamine 3000 reagent Life Technologies, Carlsbad, CA, USA) according to the manufacturer’s instructions. Viral particles were collected 72 h after transfection, filtered through a 0.45 μM membrane filter (Jet Biofil, Guangzhou, China), concentrated through an Amcon Ultra-15 Centrifugal Filter Tube (Merck Millipore), and centrifuged at 4000 xg for 30 min at 4 °C. Before transduction, the medium was changed to IMDM supplemented with 2% FBS. One hundred μl of concentrated viral particles was mixed with Polybrene Infection/Transfection Reagent (Sigma-Aldrich) at a final concentration of 8 μg/ml and then dropped into a 24-well plate containing 500,000 cells/ml. The plate was then centrifuged at 2,250 rpm for 2 h at 25 °C. The medium was changed to erythroid differentiation medium after 24 h post-transduction. Transduced cells harboring viruses containing the puromycin-resistant gene were selected after culturing in 1 μg/ml puromycin for 2 days.

### Overexpression of YAP and TAZ in CD34^+^ HSC-derived erythroid cells

The overexpressing plasmids pBEBE-Puro-Flag-YAPS5A and pBEBE-Puro-Flag-TAZS89A were kindly provided by Dr. Siew Wee Chan of the Institute of Molecular and Cell Biology, A*STAR, Singapore, as described previously [25, 26]. These plasmids were mutagenized at 5 phosphorylation sites, from serine to alanine on YAP (S61A, S109A, S127A, S164A, and S381A) designated YAPS5A, and TAZ (S89A) named TAZS89A, which resulted in YAP and TAZ proteins that are always active. Cells were transfected with the YAPS5A or TAZS89A plasmid by nucleofection.

### Knockdown of YAP and TAZ in CD34^+^ HSCs

Fresh CD34^+^ HSCs were expanded in HSPC cytokine-rich medium as published previously [27] for 4–5 days. *YAP* and *TAZ* genes were knocked down using the CRISPR/Cas9 lentiviral vectors pLenti-Puro_gYAP (GenScript, gRNA sequence: 5′-GCAGTCGCATCTGTTGCTGC-3′) and pLenti-Puro_gWWTR1 (TAZ), as described above. The puromycin-resistant CD34^+^ HSCs were mixed with MethoCult-enriched methylcellulose (H4435; STEMCELL Technologies) and cultured for 14 days.

### Cytospin and Wright’s staining

Cells were collected and spun onto a glass slide at 1000 rpm for 5 min using a Cytospin 4 (Thermo Fisher Scientific, Waltham, MA, USA). Wright’s staining solution (1.5 ml) was dropped onto the glass slide followed by 1.5 ml of distilled water and incubated for 4 min. The slide was washed with water and air dry. Permount mounting medium (Fisher Chemical, Hampton, NH, USA) was applied and coverslipped. Cell morphology was observed by a light microscope (Olympus Microscope CX31; Olympus, Tokyo, Japan).

### Western blotting analysis

Cells were collected, washed with phosphate-buffered saline (PBS), and lysed by adding radioimmunoprecipitation assay (RIPA) buffer (Thermo Fisher Scientific) containing Proteinase K (Thermo Fisher Scientific) and phosphatase inhibitors (Sigma-Aldrich), incubated on ice for 30 min. The protein concentrations were measured using a Pierce BCA Protein Assay Kit (Thermo Fisher Scientific). Protein was boiled, loaded onto 7.5–12% sodium dodecyl sulfate–polyacrylamide gel electrophoresis (SDS-PAGE) and transferred to a 0.45 μM polyvinylidene fluoride (PDVF) membrane (Merck Millipore). The membrane was blocked with 5% skimmed milk (Merck Millipore) in Tris-buffered saline containing 0.1% Tween-20 for 1 h at room temperature (RT) and probed with the primary antibodies: TAZ (#4883; Cell Signaling Technology (CST), Danvers, MA, USA), YAP (#4912; CST), pTAZ (S89) (#75275; CST), pYAP (S127) (#4911; CST), LATS1 (#9153; CST), LATS2 (#5888; CST), pLATS-1079 (#8654; CST), pLATS-909 (#9157; CST), and Caspase3 (#9665; CST) diluted at 1:1,000, overnight at 4 °C. The membrane was then incubated with HRP-conjugated secondary antibody diluted at 1:5,000 for 2 h at RT followed by staining with HRP-conjugated anti-human β-actin (1:10,000) for 30 min. The membrane was incubated with enhanced chemiluminescence (ECL) (Bio-Rad Laboratories, Hercules, CA, USA) and visualized using a biomolecular imager (ImageQuant LAS4000; GE Healthcare, Chicago, IL, USA).

### RNA isolation and gene expression analysis

RNA was prepared using TRIzol reagent (Molecular Research Center, Cincinnati, OH, USA). Total RNA 500 ng, measured using a Nanodrop 2000 spectrophotometer (Thermo Fisher Scientific), was reverse-transcribed using a ReverseAid First Strand cDNA Synthesis Kit (Thermo Fisher Scientific). Quantitative real-time reverse transcription-polymerase chain reaction (qRT-PCR) was performed using TaqMan Fast Universal PCR Master Mix (Applied Biosystems, Foster City, CA, USA) and the Universal Probe Library (UPL; Roche Life Science, Penzberg, Germany) on a CFX356 Real-Time PCR Detection System (Bio-Rad Laboratories). Data were normalized to *GAPDH*. Primer sequences and probes are listed in Additional file 1: Table S2.

### Apoptosis assay

Cells were harvested, resuspended in 100 μl of 1 × binding buffer containing 5 μl Annexin V-FITC (BD Bioscience, San Jose, CA, USA) and 5 μl 7-AAD (BD Bioscience), and incubated for 15 min at RT in the dark. Binding buffer was added up to 300 μl, and cells were analyzed by flow cytometry.

### Flow cytometry analysis

Cells were harvested, washed with PBS twice, and incubated with the desired antibodies for 15 min at RT in the dark. Cells were washed twice, centrifuged at 2000 rpm, and resuspended in 300 μl of 2% FBS/PBS before being subjected to FACSCanto flow cytometry (BD Bioscience) and analyzed using FACS DIVA software (BD Bioscience). The antibodies used for flow cytometry were anti-human CD34-PE (#343506; BioLegend, San Diego, CA, USA), anti-human CD235a-APC (#130-100-270; Miltenyi Biotec), and anti-human CD41-FITC (#303704; BioLegend).

### Statistical analysis

Results are expressed as mean ± standard error of the mean (SEM). The significant difference between groups was analyzed using non-parametric Mann–Whitney test or Kruskal–Wallis test for experiment that has two groups or more than two groups, respectively. Analysis was performed on GraphPad Prism software version 8 for Mac (GraphPad Software, San Diego, CA, USA). A *p *value < 0.05 was statistically significant.

## Results

### YAP and TAZ are highly expressed in pro-erythroblasts and erythroblasts

**Fig. 1:**
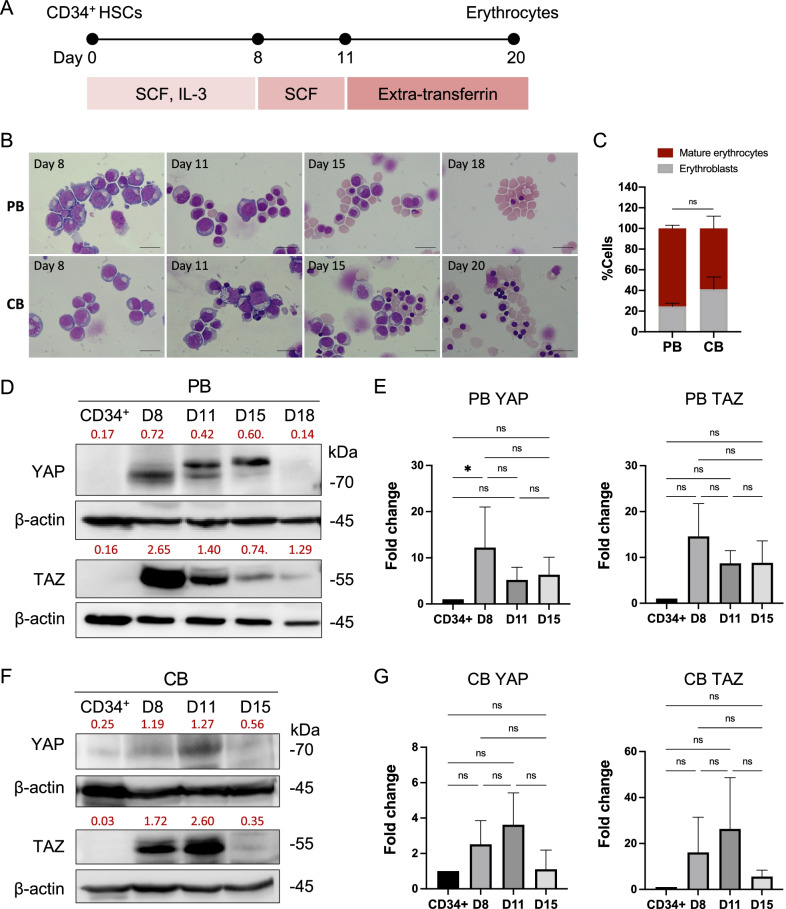
Expression of YAP and TAZ in CD34^+^ HSC-derived erythroblasts. **A** Schematic of erythroid differentiation from human CD34^+^ HSCs in the three-stage erythroid culture system. **B** Wright’s staining of erythroid cells during differentiation of mobilized-peripheral blood (PB)- and cord blood (CB)-CD34^+^ HSCs showing the morphologic change from CD34^+^ HSC to pro-erythroblasts on day 8, erythroblasts on days 11–15, and mature erythrocytes on days 18 and 20 in in vitro culture, respectively. **C** Percentage of mature erythrocytes derived from PB- and CB-CD34^+^ HSCs in the three-stage erythroid culture system at the end of culture. At least 500 cells were counted in each group (*n* = 4). **D**, **F** Western blot analysis of total YAP and TAZ expression at days (**D**) 8, 11, 15, and 18 of erythroid-differentiated cells derived from PB- and CB-CD34^+^ HSCs, respectively. Relative expression levels of YAP and TAZ to β-actin as measured by Image J software were labeled in red (PB and CB, *n* = 5, 3, respectively). **E**, **G** Fold intensity of YAP and TAZ were analyzed compared to CD34^+^ cells. Data represent the mean ± standard error of the mean (SEM), *p < 0.5. Scale bar, 20 μm

Human CD34^+^ HSCs—from mobilized peripheral blood (PB) and cord blood (CB), were collected. A purity of CD34^+^ HSCs greater than 95%, as analyzed by flow cytometry, was used in all experiments. CD34^+^ HSCs were differentiated to erythrocytes by culturing in a three-stage erythroid culture system [22] (Fig. 1A). During erythroid differentiation, the cultured CD34^+^ HSCs exhibited morphology resembling pro-erythroblasts, basophilic erythroblasts, polychromatic erythroblasts, orthochromatic erythroblasts, and mature erythrocytes (Fig. 1B). With this culture system, CD34^+^ HSCs derived from PB showed morphological differentiation sooner and generated a higher percentage of enucleated mature erythrocytes at the end of culture than those derived from CB. Although not significant difference, the percentage of mature erythrocytes derived from PB- and CB-CD34^+^ HSCs at the end of culture was 75% (day 18) and 60% (day 20), respectively (Fig. 1C).

To determine basal expression of YAP and TAZ in human erythropoiesis, protein expression of YAP and TAZ in HSCs and differentiated erythroid cells from culture on days 8, 11, 15, and 18 was studied by Western blot. The expression of YAP and TAZ protein was barely detected in PB- and CB-CD34^+^ HSCs. In contrast, the expression of these two proteins became highly upregulated in cultured cells on days 8 and 11 when the majority of cells became pro-erythroblasts and erythroblasts and it trend to be downregulated as erythroblasts undergo maturation and enucleation, but not statically significant (Fig. 1D–G). Furthermore, the highest expression of YAP/TAZ in erythroid cells of PB origin was on day 8, while the highest expression of YAP/TAZ in cells of CB origin was on day 11. This might be because erythroid cells from CB demonstrate slower morphological change compared to those from PB (Fig. 1B), which resulted in YAP/TAZ in cells of CB origin becoming upregulated later. In addition, phosphorylated (p)YAP and pTAZ (the inactive forms of YAP and TAZ) and their upstream mediators (LATS1, LATS2, and pLATS proteins) were also detected during erythroid differentiation of PB-HSCs (Additional file 1: Fig. S1). Overall, our result showed that YAP and TAZ are highly expressed in pro-erythroblasts and erythroblasts compared to the non-differentiated CD34^+^ HSCs of both fetal and adult origin, which suggests that YAP/TAZ might play a crucial role in human erythropoiesis.

### Inhibition of YAP/TAZ activity inhibited erythroid cell proliferation and induced cell apoptosis at a late stage of erythroid differentiation

To determine whether YAP/TAZ is required for erythroid differentiation, we modulated the expression of YAP/TAZ using two small molecules, lysophosphatidic acid (LPA), and dobutamine hydrochloride (DH). LPA has been classified as a YAP/TAZ activator, which increases YAP/TAZ activity by enhancing their nuclear localization and interaction with their DNA binding partner TEAD1 [28–30]. In contrast, DH is a YAP/TAZ inhibitor, increases phosphorylation, and prevents their nuclear localization [19]. We first evaluated the effect of LPA and DH on proliferation rate of PB-CD34^+^ HSCs in the erythroid culture system, and we determined a concentration of 10 μM to be the optimal dose for both agents (Additional file 1: Fig. S2).

**Fig. 2:**
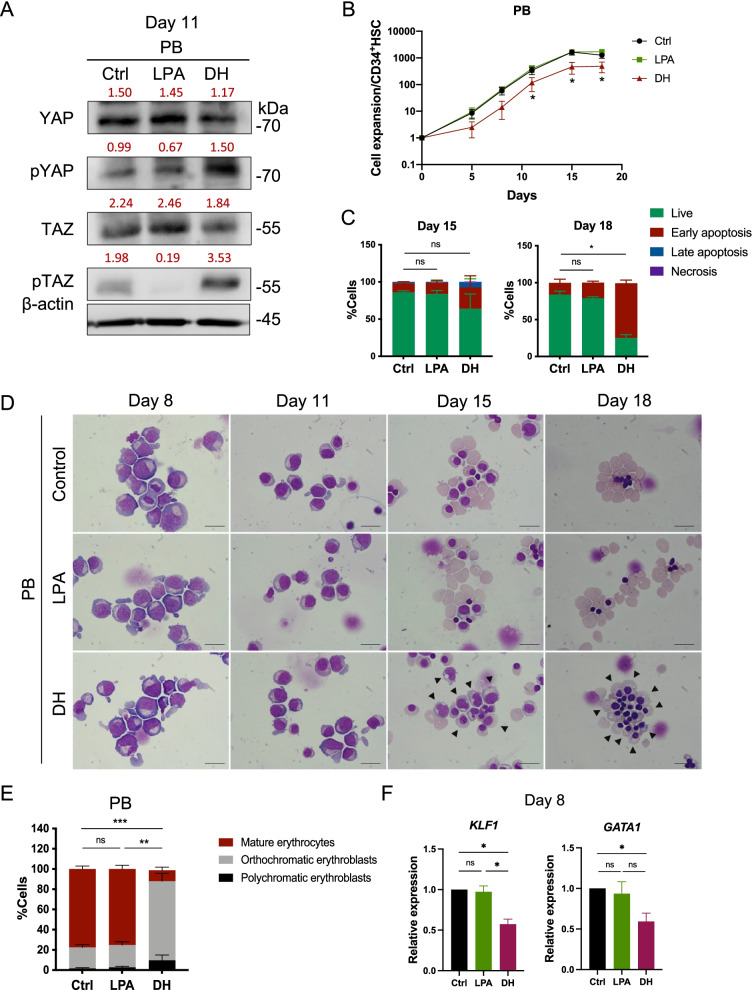
Depletion of YAP/TAZ protein by DH-impaired erythroid cell proliferation and maturation. **A** Expression of YAP and TAZ after 10 μM lysophosphatidic acid (LPA; a YAP/TAZ activator) and dobutamine hydrochloride (DH; a YAP/TAZ inhibitor) treatment of PB-CD34^+^ HSC-derived erythroblasts for 11 days. Relative levels to β-actin were labeled in red. **B** Fold increase of cells during erythroid differentiation from PB-CD34^+^ HSCs after treatment with LPA (green) and DH (red) when compared to control (black) (*n* = 5). **C** Cell apoptosis of PB-CD34^+^ HSC-derived erythroid cells at days 15, and 18 after LPA and DH treatment, as analyzed by Annexin V and 7-AAD staining (*n* = 3). **D** Representative cell morphology during erythroid differentiation from PB-CD34^+^ HSCs showed morphological delay around day 15, and most of the remaining cells were erythroblasts (black arrow) at day 18 after DH treatment. **E** Percentage of mature erythrocytes and erythroblasts at the terminal stage of differentiation (day 18). At least 500 cells were counted in each group (*n* = 9). **F** Expression of the erythroid-specific genes *KLF1* and *GATA1*, after LPA and DH treatment for 8 days (*n* = 3). Data represent the mean ± SEM, *p < 0.5, **p < 0.01, ***p < 0.001. Scale bar, 20 μm

To determine the effect of LPA and DH on YAP/TAZ protein expression during erythroid differentiation from HSCs, PB-, and CB-CD34^+^ HSCs were cultured in the three-stage erythroid culture system supplemented with either 10 μM LPA or 10 μM DH. Cell pellet of LPA and DH treatment is shown in Additional file 1: Fig. S3. Differentiating cell at day 11 of culture (most cells are erythroblasts) was collected for Western blot analysis. Result showed that LPA slightly increased TAZ proteins expression, while DH increased pYAP and pTAZ of PB-HSC derived erythroblasts when compared with control (Fig. 2A). To confirm that LPA and DH could indeed manipulate YAP/TAZ activity, expression of YAP/TAZ target genes: *c-Myc*, *CTGF*, *Cyclin D1* and *CYR61* were determined (Additional file 1: Fig. S4). Result showed that LPA slightly increased YAP/TAZ activity in this culture system, but DH clearly inhibited YAP/TAZ activity as shown by the significant down-regulation of their target genes.

We next determined the growth kinetics of differentiated PB-HSCs during erythroid culture in LPA and DH treatment. The results showed that LPA at a concentration of 10 μM did not affect the growth kinetics of erythroid cells during the differentiation of PB-HSCs. In contrast, DH has a significantly negative effect on the growth kinetics of PB-HSCs (Fig. 2B), either by inhibiting cell proliferation or by inducing cell apoptosis. Subsequent apoptosis assays of PB-CD34^+^ HSC-derived erythroid cells showed that although DH did not induce cell apoptosis on culture day 11 (data not shown), its effects started to induce cell apoptosis on culture day 15, and more significantly on day 18. In contrast, LPA treatment did not affect cell apoptosis (Fig. 2C). These results suggest that the inhibition of YAP/TAZ by DH did not induce cell apoptosis at an early stage of differentiation but rather inhibits erythroid cell proliferation or promotes maintenance of cell quiescence. Increased cell apoptosis of PB-CD34^+^ HSC-derived erythroid cells occurred only during the late stages of erythroid differentiation, possibly by increasing caspase-3 activity (Additional file 1: Fig. S5). Inhibition of cell proliferation might lead to the later activation of an apoptotic pathway in this scenario.

### Inhibition of YAP/TAZ proteins by DH-impaired erythroid cell differentiation

We next determined the effect of YAP/TAZ on erythroid differentiation. LPA treatment had no apparent additional positive effect on erythroid differentiation of PB-CD34^+^ HSCs. However, DH treatment delayed differentiation and impaired enucleation of PB-HSC-derived erythroblasts, as determined by morphological changes during culture days 15 to 18 (Fig. 2D). The percentage of enucleated mature erythrocytes at the end of culture was decreased dramatically in the DH-treated group compared to the control (Fig. 2E). In addition, the erythroid-specific genes *KLF1* and *GATA1* were downregulated in DH-treated erythroblasts derived from PB-CD34^+^ HSCs (Fig. 2F*)*. Erythroid differentiation of CB-CD34^+^ HSCs showed cell proliferation and differentiation impairment similar to that observed in PB-CD34^+^ HSCs, but there appeared to be less effective after DH treatment (Additional file 1: Fig. S6A–D*)*.

### Interaction of YAP/TAZ-TEAD is required during erythroid differentiation

**Fig. 3:**
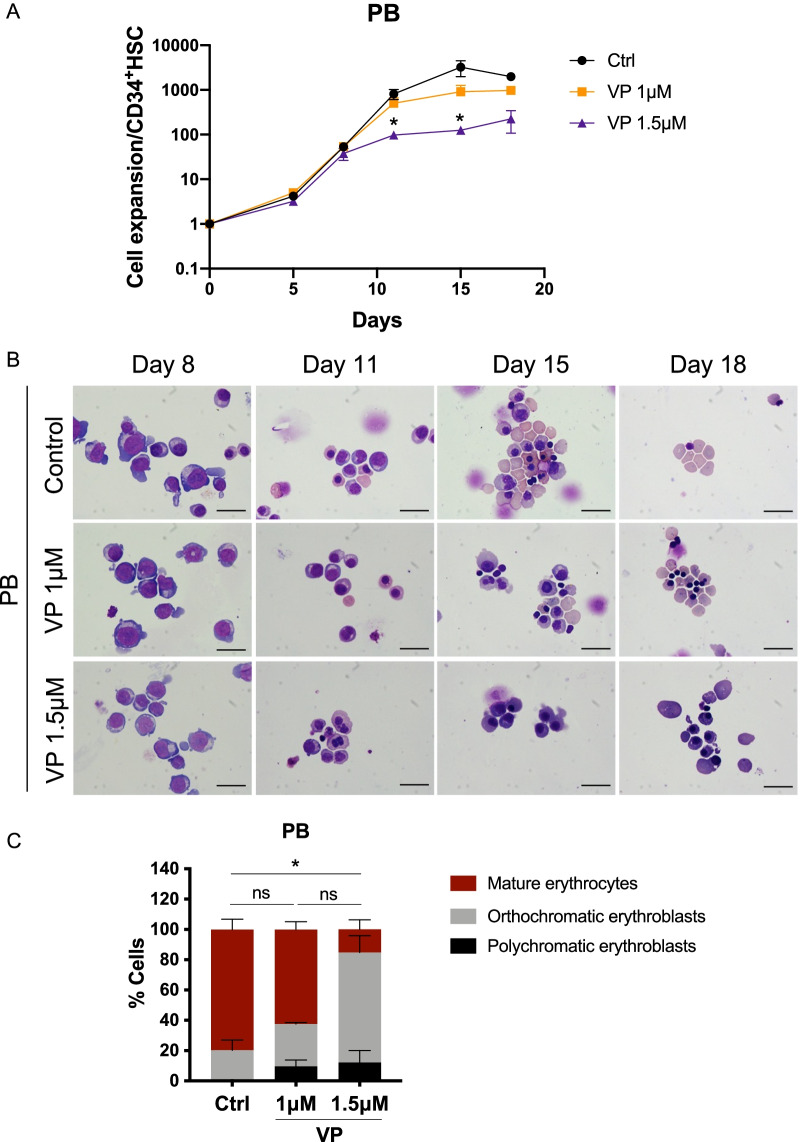
Inhibition of YAP/TAZ-TEAD interaction by verteporfin impaired erythroid differentiation. **A** Fold increase of PB-CD34^+^ HSC-derived erythroid cells after treatment with verteporfin (VP) at 1.0 μM (orange) and 1.5 μM (purple) compared to control (black) (*n* = 3). **B** Representative cell morphology during erythroid differentiation after VP treatment. **C** Percentages of mature erythrocytes and erythroblasts on day 18. At least 500 cells were counted in each group (*n* = 3). Data represent the mean ± SEM, *p < 0.5. Scale bar, 20 μm

We used another small molecule, verteporfin (VP), that inhibits the interaction between YAP and its transcription factor TEAD by changing the conformation of YAP and increasing its degradation [20, 21, 31]. Similar to DH treatment, VP inhibited the growth kinetics and the erythroid differentiation of PB-CD34^+^ HSCs, as demonstrated by the low number of mature erythrocytes at the final stage of erythroid culture in a dose-dependent manner (Fig. 3A–C). Taken together, inhibiting the active form of YAP/TAZ co-transcription factor activity via phosphorylation or by preventing the formation of YAP/TAZ-TEAD binding to DNA impairs erythroid differentiation.

### DH treatment specifically impaired erythroid maturation and enucleation from erythroblasts to erythrocytes

**Fig. 4:**
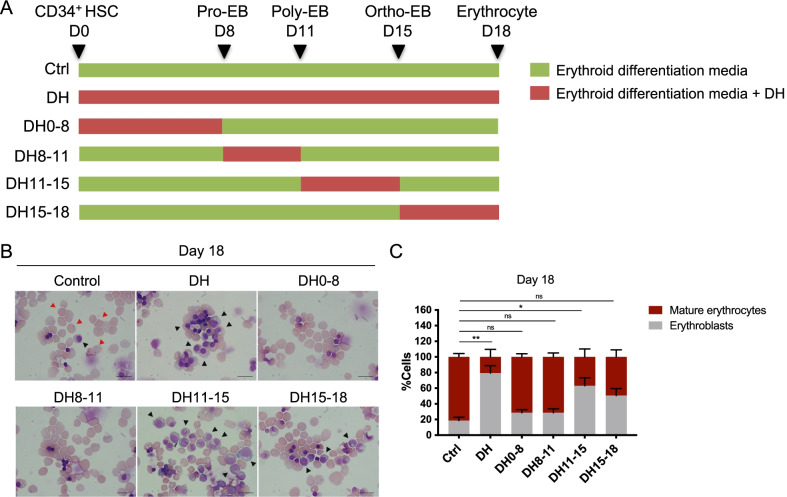
DH treatment specifically impaired erythroid maturation and enucleation from erythroblasts to erythrocytes. **A** Schematic of DH treatment at various time points during erythroid differentiation. **B** Representative of erythroid cell morphology of differentiated cells at day 18 following treatment with DH at the various time points described above, showing mature erythrocytes (red arrows) and erythroblasts (black arrows). **C** Percentages of mature erythrocytes and erythroblasts after timed DH treatment during differentiation at the terminal differentiation stage (day 18). At least 500 cells were counted in each group (*n* = 4). Data represent the mean ± SEM, *p < 0.5, **p < 0.01. Scale bar, 20 μm

To determine the exact time points at which DH affects erythroid differentiation, we initiated DH treatment at various specific time points of culture (Fig. 4A). Differentiating PB-HSCs that received DH treatment while differentiating to erythroid cells only during culture days 11–15 (DH11-15) showed signs of impaired erythroid maturation in a manner similar to those that received DH treatment throughout the entire culture period (DH) (Fig. 4B, C). In contrast, the PB-CD34^+^ HSC-derived erythroid cells that received DH treatment at other time points, such as during culture days 0–8 (DH0-8), and during culture days 8–11 (DH8-11), showed no significant signs of impaired erythroid maturation, while those treated during culture days 15–18 (DH15-18) showed only minor effects of erythroid enucleation impairment. These results suggest that the inhibitory effect of DH on erythroid differentiation occurred mainly at the onset of erythroblast conversion to erythrocyte.

### Knockdown of YAP and TAZ genes impaired human erythroid maturation

**Fig. 5:**
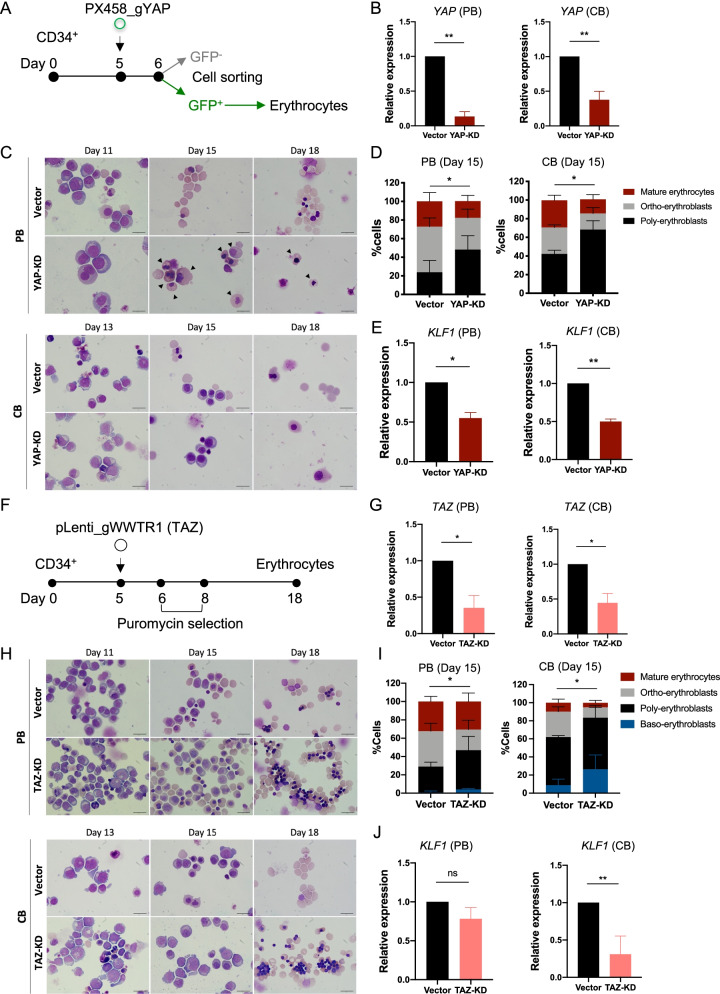
YAP and TAZ knockdown delayed erythroid maturation and enucleation. **A** Schematic of YAP knockdown during erythroid differentiation using the CRISPR/Cas9 PX458-GFP_gYAP plasmid. **B** *YAP* mRNA expression of PB- and CB-CD34^+^ HSC-derived erythroblasts at 3 days post-nucleofection, as analyzed by qRT-PCR (*n* = 4). **C** A representative of erythroid cell morphology during differentiation after YAP-KD showed delayed differentiation and more immature cells (black arrow) compared to control. **D** Percentages of cells after YAP-KD of PB- and CB-CD34^+^ HSCs counted at day 15 of differentiation. At least 200 cells were counted in each group (*n* = 4). **E** *KLF1* mRNA expression of sorted GFP^+^ cells at 3 days after nucleofection (*n* = 3). **F** Schematic of TAZ knockdown during erythroid differentiation using CRISPR/Cas9 pLenti_gWWTR1 (TAZ). **G** *TAZ* mRNA expression at 3 days post-nucleofection (*n* = 3, 4, respectively). **H** Representative erythroid cell morphology derived from PB- and CB-CD34^+^ HSCs, after TAZ-KD. **I** Percentages of cells after TAZ-KD at day 15 of differentiation. At least 200 cells were counted in each group (*n* = 3, 4, respectively). **J** *KLF1* mRNA expression after TAZ-KD analyzed 3 days after nucleofection (*n* = 3). Data represent the mean ± SEM. *p < 0.05, **p < 0.01. Scale bar, 20 μm

To confirm the role of YAP on human erythroid differentiation, HSC-derived erythroblasts were genetically manipulated using CRISPR/Cas9 plasmids containing a single guide RNA (sgRNA) targeting YAP. Three gRNA sequences were designed to target YAP at exon 1 using CRISPR.mit.edu (Additional file 1: Fig. S7A). gRNA number 2 was chosen for further experiments based on its knockdown efficiency in HEK293FT cells (Additional file 1: Fig. S7B, C), and it was designated PX458-GFP_gYAP. This vector has previously been used for the generation of YAP-depletion iPS cell line [32]. To knock down YAP, the PX458-GFP_gYAP plasmid was transfected into HSC-derived erythroid cells on culture day 5 (Fig. 5A), and the successfully transformed HSC-derived erythroblasts that expressed GFP (Additional file 1: Fig. S7D–F) were sorted by FACS and further cultured in erythroid culture system for subsequent use in the erythroid differentiation assay. Although our gene inactivation approach did not completely abolish *YAP* mRNA expression, its expression in YAP-KD PB- and CB-HSCs was significantly downregulated (Fig. 5B) and YAP target genes: *c_Myc*, *Cyclin D1* and *CYR61* were significantly decreased (Additional file 1: Fig. S8). Similar to DH and VP treatment, YAP-KD HSC-derived erythroid cells failed to complete erythroid differentiation and enucleation. We observed that YAP-KD cells demonstrated delayed erythrocyte morphology at day 15. They started to die before enucleation took place. However, in the vector control (PX458-GFP), cells enucleated usually on day 18 (Fig. 5C). The higher percentages of immature polychromatic erythroblasts on day 15 suggest that the polychromatic erythroblasts derived from YAP-KD cells experienced developmental arrest and could not further differentiate to become mature erythrocytes (Fig. 5D). In addition to their erythroid differentiation defects, the YAP-KD cells also expressed significantly lower *KLF1* erythroid-specific genes (Fig. 5E).

Since TAZ shares partial functional redundancy with YAP in organ growth control, we investigated whether or not TAZ has a similar role in erythroid differentiation. To that end, we knocked down the *TAZ* gene using the pLenti-Puro_gWWTR1 (TAZ) plasmid, which was previously used for the generation of TAZ-depleting iPSCs [33]. This plasmid was transduced into HSC-derived erythroid cells on culture day 5, and successfully transduced HSCs that were resistant to puromycin were identified and evaluated in erythroid differentiation assay (Fig. 5F). The expression level of *TAZ* mRNA in TAZ-KD PB- and CB-HSCs was significantly lower than in those cells transduced with a control plasmid (Fig. 5G). Nonetheless, a phenotypic delay was observed in TAZ-KD cells (Fig. 5H). More specifically, the ratio of polychromatic and orthochromatic erythroblasts differed on day 15 of culture compared to control, suggestive of erythroid differentiation delay (Fig. 5I). These cells of CB-HSC origin also expressed a significantly lower level of *KLF1* mRNA, but it was only somewhat reduced in cells of PB-HSC origin (Fig. 5J). Furthermore, these data suggest that TAZ-KD cells show differentiation delay, similar to YAP-KD cells, which indicates that YAP and TAZ may be required for normal erythroid differentiation to the terminal stage.

### Overexpression of YAP/TAZ exerted no effect on erythroid differentiation

**Fig. 6:**
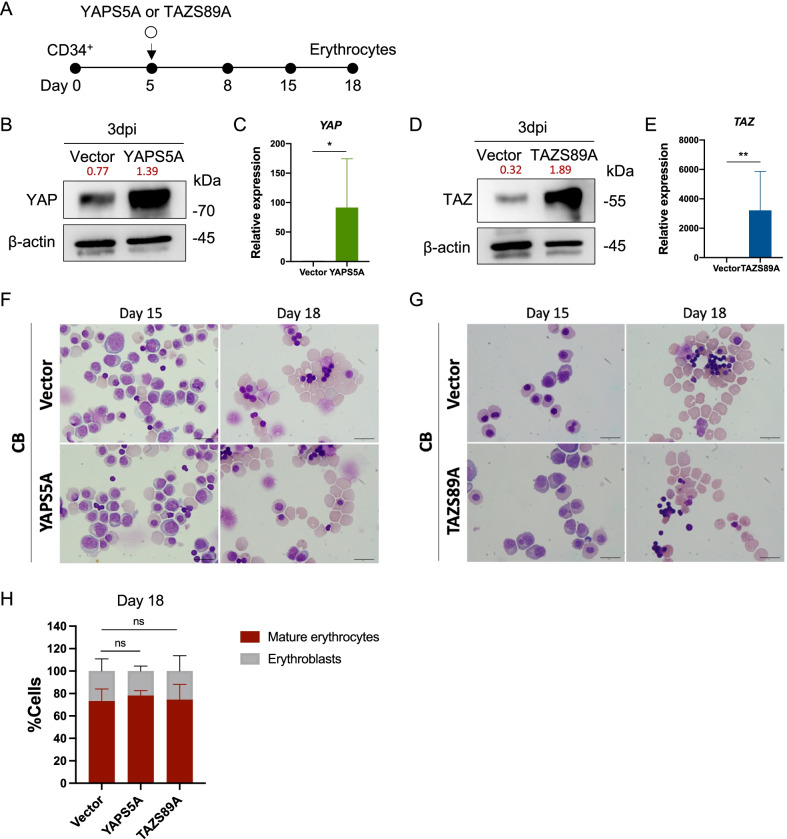
Overexpression of YAP/TAZ had no apparent effect on erythroid differentiation. **A** Schematic of YAP and TAZ overexpression in erythroid cells using constitutively active YAP and TAZ plasmids named YAPS5A and TAZS89A, respectively. **B** YAP protein expression analyzed by Western blotting at 3 days post-nucleofection and relative expression to β-actin were labeled in red. **C** *YAP* mRNA expression as analyzed by qRT-PCR at 3 days post-nucleofection (*n* = 4). **D** TAZ protein expression as analyzed by Western blotting at 3 days post-nucleofection and relative expression to β-actin were labeled in red. **E** *TAZ* mRNA expression was analyzed by qRT-PCR at 3 days post-nucleofection (*n* = 5). **F**, **G** Representative cell morphology after YAP and TAZ overexpression. **H** Percentages of cells after YAP and TAZ overexpression at day 18 of differentiation. At least 200 cells were counted in each group (*n* = 3). Data represented in the mean ± SEM. *p < 0.05, **p < 0.01. Scale bar, 20 μm

As shown in the earlier experiments, increasing of YAP activity using LPA treatment did not show significant effect on erythroid cell growth. Since increasing of LPA concentration is not doable in this circumstance due to its cytotoxic effect, we then performed a genetic manipulation experiment by transducing constitutively active YAP and TAZ plasmids containing phosphorylated sites mutated at serine →alanine, namely YAPS5A and TAZS89A, respectively, to the CB-CD34^+^ HSC-derived erythroid cells on culture day 5 (Fig. 6A). Both qRT-PCR and Western blot analysis confirmed dramatically increased total YAP/TAZ expression in transformed cells at 3 days post-transduction and increasing of their target gene *c-Myc* was observed (Fig. 6B–E, Additional file 1: Fig. S9). We found that overexpression of YAP/TAZ did induce erythroid-specific genes upregulation; *KLF1, GATA1* and *TAL1* (Additional file 1: Fig. S10). However, corresponding to the result from LPA treatment, we did not observe any apparent beneficial effect of erythroid differentiation after YAP/TAZ overexpression as determined by cell morphology when compared to control (Fig. 6F–H). Altogether, these results suggested that there might be a limited threshold level of YAP in erythroid cell differentiation process. Increasing YAP activity over the threshold has no additional beneficial effect on human erythroid cells development.

### YAP and/or TAZ are dispensable during human HSC lineage allocation to myeloid or erythroid lineages

**Fig. 7:**
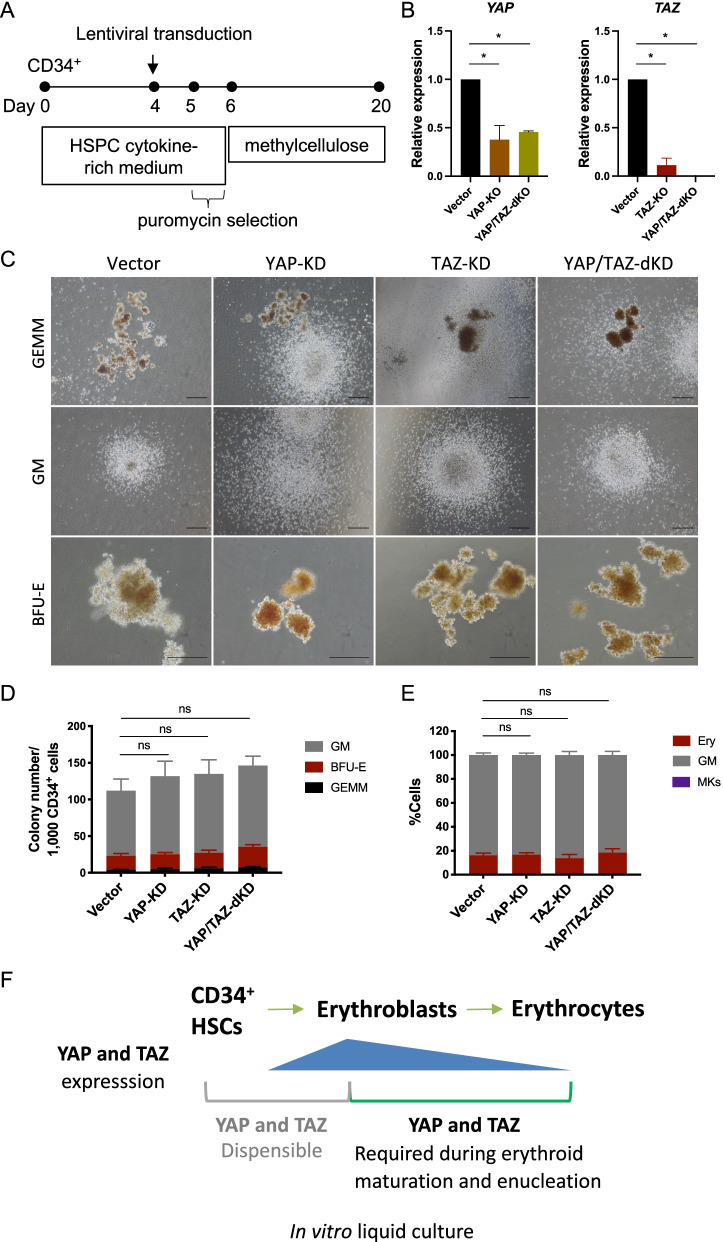
Knockdown of YAP or TAZ in CD34^+^ HSCs did not alter HSC commitment to erythroid or myeloid lineages. **A** Schematic of YAP and TAZ knockdown in CD34^+^ HSCs using CRISPR/Cas9 lentiviral system. **B** *YAP* and *TAZ* mRNA expression as analyzed by qRT-PCR at 2 days after lentiviral transduction (*n* = 4, 3 respectively). **C** Representative of colony-forming unit (CFU) assay of YAP-KD, TAZ-KD, and YAP/TAZ-double (d) KD in PB-CD34^+^ HSCs after being cultured in methylcellulose for 14 days showing colony-forming unit granulocyte/erythroid/macrophage/megakaryocyte (CFU-GEMM), colony-forming unit granulocyte/macrophage (CFU-GM) and Burst-forming unit erythroid (BFU-E). **D** The colony number of YAP-KD, TAZ-KD, and YAP/TAZ-dKD derived from PB-CD34^+^ HSCs counted on day 14 of methylcellulose culture (*n* = 3). **E** Percentage of erythroid cells (Ery) and granulocytes/macrophages (GM) after YAP-KD, TAZ-KD, and YAP/TAZ-dKD of cells from methylcellulose (*n* = 3). **F** Summary of this study. Data represent the mean ± SEM, *p < 0.05. Scale bar, 200 μM

To determine whether or not YAP/TAZ is required during the establishment of erythroid or myeloid lineages from HSCs to their progenitors, CD34^+^ HSCs were cultured in HSPC cytokine-rich medium for pre-activation and expansion [27]. CD34^+^ HSCs were transduced using lentiviral particles containing a single-guide RNA CRISPR/Cas9 targeted to YAP or TAZ (pLenti_Puro_gYAP and pLenti_Puro_gWWTR1 [TAZ], respectively), followed by puromycin selection (Fig. 7A). Puromycin-resistant HSCs were seeded into methylcellulose for colony-forming unit assay which is a standard testing of CD34^+^ HSCs differentiation ability to their progenitors [34, 35]. Methylcellulose supports colony-forming unit granulocytes, erythroid, megakaryocyte, and macrophage (CFU-GEMM, multi-lineage progenitors); colony-forming unit granulocyte/macrophage (CFU-GM, granulocyte and macrophage progenitors); and erythroid (BFU-E, CFU-E, erythroid progenitor). Although the expression of YAP/TAZ in HSCs was barely detectable by Western blot analysis (Fig. 1D, F), it could be detected by RT-qPCR (Fig. 7B). The expression of *YAP* and *TAZ* mRNA was significantly reduced in YAP-KD, TAZ-KD, and YAP/TAZ-double (d) KD compared to control (empty vector without gRNA) (Fig. 7B). After 14 days of culture, we found that all colony types could be observed in the empty vector control, YAP-KD, TAZ-KD, and YAP/TAZ-dKD (Fig. 7C). The number of each colony after YAP-KD, TAZ-KD, and YAP/TAZ-dKD did not significantly differ from the control (Fig. 7D). We further collected all cells from methylcellulose culture and stained them for specific lineage markers: Glycophorin A (GPA, also known as CD235a), erythroid lineage marker and CD41, megakaryocyte lineage marker. We used GPA^−^CD41^−^ to classify granulocyte/macrophage population in this study (Additional file 1: Fig. S11). The proportion of granulocyte/macrophage lineage and erythroid lineage did not significantly differ after YAP-KD, TAZ-KD, or YAP/TAZ-dKD compared to control (Fig. 7E). These results indicate that loss of YAP and TAZ expression does not alter HSC differentiation to myeloid or erythroid progenitors and suggests that they might not be involved in HSC lineage allocation to myeloid-erythroid lineages in this culture system. Summary of this study is illustrated in Fig. 7F.

## Discussion

Our results provide the clear evidence that YAP and TAZ are required for human terminal erythroid differentiation and enucleation in both fetal and adult HSCs, but that they are dispensable for erythroid lineage establishment from HSCs. YAP and TAZ are probably required for human erythroid cell proliferation and protects these cells from apoptosis, similar to Hippo pathway-regulated cell growth in other cell types [36]. However, expression of YAP/TAZ is an absolute requirement for the successful transition of erythroblast maturation to erythrocyte.

Although, previous studies reported that YAP is dispensable for normal HSC function [37, 38] and overall blood production, including erythrocytes, in normal and malignant hematopoiesis using the conditional inducible YAP/TAZ depletion mice model under the *Mx1-Cre* promoter [39]. However, these results do not completely abnegate our results as the fundamental differences between human and mouse erythropoiesis have long been demonstrated. Although mouse has extensively been used as a model for improving our understanding of the mammalian erythropoiesis. Nonetheless, there are several variations between human and mouse hematopoiesis, especially at the terminal erythroid differentiation stage. An and colleagues (2014) performed transcriptomic analysis on the isolated pure population of human and mouse erythroblasts. The analysis of transcriptomic data revealed that there were significant stage and species-specific differences of terminal erythroid differentiation [40]. In addition, the differences on expression of long-non-coding RNA and other erythroid cell maturation related genes between human and mouse were also demonstrated [41–43]. These differences highlight the complications in translating observations from mice to human and shed light onto why some human hematologic disorders are not knowledgeable in mouse models.

It has also been reported that YAP/TAZ expression in vivo could be altered by negative feedback or redundancy by other factors. Indeed, a negative feedback loop to inhibit YAPS5A, overexpression of YAP activity that translocates into a nuclease, such as α-catenin, was found in cardiomyocytes [44] and epidermal cells [45]. Furthermore, during differentiation of mouse embryonic stem cells to macrophages, YAP-TEAD seems to have a role in hemogenic endothelium transition stage [46] and regulate the HSPC formation in zebrafish involved in mechanosensing [47]. Recent finding has also demonstrated that Yap1 promotes proliferation of stress erythroid progenitors during recovery from bone marrow transplantation in mice [48]. It might suggest that YAP is required for HSC formation from hemogenic endothelial transition stage but dispensable for establishment to its progenitors such as erythroid and myeloid progenitors. However, YAP is required again during erythrocyte maturation and enucleation. It indicates the dynamic of YAP during development.

It might be possible that the YAP/TAZ-TEAD complex controls the expression of erythroid specific genes *KLF1* and *GATA1* as the expressions of *KLF1* and *GATA1* are correlated with YAP/TAZ expression and knockdown of *KLF1* and *GATA1* showed some overlapping phenotypes. Downregulation of *KLF1* led to impaired differentiation and enucleation of hESC-derived erythroid cells [49], whereas reduction of *GATA1* inhibited erythroid differentiation, which resulted in the development of immature erythroid cells [50].

YAP and the Hippo pathway are tightly regulated by post-translational regulators. YAP activity is controlled by LATS kinases, which are known as crucial upstream YAP inactivation. LATS kinases inhibit YAP by phosphorylation, which leads to cytoplasm retention and degradation. In addition to inhibition of YAP by phosphorylation, LATS have been reported to have the ability to translocate directly into the nuclease and phosphorylated CCCTC-binding factor (CTCF), a nuclear protein, which leads to a change in chromatin structure encompassing YAP target genes resulting in downregulation of YAP target genes [51]. Well-known upstream mediators of the Hippo pathway are controlled by cell–cell contact [52]. Recently, YAP and TAZ could be regulated by mechanical cues, including ECM stiffness, shear force, and cytoskeleton tension [53]. Furthermore, a new regulator of Hippo pathway/YAP was recently discovered via mechanotransduction and shear force through caveolae, which are organelles localized on the plasma membrane [54, 55]. In addition, YAP regulation of HSC formation in response to biomechanical force has been reported [47]. However, other types of upstream signaling and control of Hippo pathway cascades remain largely unknown.

## Conclusions

Our results demonstrated that dynamic expressions of YAP and TAZ are crucial for human erythropoiesis. The presence of YAP/TAZ essentially during the transition of erythroblast to erythrocyte is required for the successful generation of mature erythrocytes and reticulocytes.

## Supplementary Information

**Additional file 1.** **Figure S1.** Expression of pYAP, pTAZ, and their upstream mediators LATS1/2 kinases during erythroid differentiation, as analyzed by Western blot analysis. **Figure S2.** Effect of LPA and DH on proliferation rate of PB- and CB- CD34^+^ HSC at various concentration. **Figure S3.** Cell pellet of PB-derived erythroid cells after 10 μM LPA and DH treatment. **Figure S4.** Expression of YAP target genes: *c-Myc, CTGF, Cyclin D1* and *CYR61* after DH and LPA treatment for 11 days of PB-HSC-derived erythroblasts. **Figure S5.** Expression of cleaved caspase 3, a pro-apoptotic protein, after treatment of PB-CD34+ HSCs with 10 μM LPA and 10 μM DH on the terminal day of differentiation (day 18). **Figure S6.** Depletion of YAP/TAZ impaired erythroid differentiation from CB-CD34^+^ HSCs similar to PB-CD34^+^ HSCs. (A) Effect of LPA and DH on YAP/TAZ expression of CB-CD34^+^ HSC-derived erythroblasts after adding 10 μM LPA or 10 μM DH every other day for 11 days. (B) Fold increase of cells during erythroid differentiation from CB-CD34+ HSCs after treatment with LPA (green), DH (red) and control (black) (n = 3). (C) Representative cell morphology during erythroid differentiation from CB-CD34^+^ HSCs after LPA and DH treatment, erythroblasts (black arrow). (D) Percentage of mature erythrocytes and erythroblasts at the terminal stage of differentiation (day 20). At least 500 cells were counted in each group (n=4). Data represent the mean ± SEM. *p<0.05, Student’s t-test. Scale bar, 20 μm.

## Data Availability

All datasets in this article are included within the article and additional files.

## Abbreviations

BFU-E: Burst-forming unit erythroid
c-Myc: Cellular myelocytomatosis
CB: Cord blood
CD: Cluster of differentiation
CFU-E: Colony-forming unit erythroid
CFU-GEMM: Colony-forming unit granulocyte/erythrocyte/monocyte/megakaryocyte
CFU-GM: Colony-forming unit granulocyte/macrophage
CRISPR: Clustered regularly interspaced short palindromic repeats
CYR61: Cysteine-rich angiogenic inducer 61
D: Day
DH: Dobutamine hydrochloride
dpi: Day post-induction/transfection
GATA1: GATA-binding transcription factor 1
GPA: Glycophorin A
h: Hour
HSCs: Hematopoietic stem cells
IL: Interleukin
KD: Knockdown
KLF1: Kruppel-like factors 1
LPA: Lysophosphatidic acid
PB: Peripheral blood
PBS: Phosphate-buffered saline
qRT-PCR: Quantitative reverse transcriptase-polymerase chain reaction
TAL1: T-cell acute lymphocytic leukemia 1
VP: Veterporfin
WWTR1: WW domain-containing transcription regulator protein 1
YAP: Yes-associated protein

## References

[1] Trakarnsanga K, Griffiths RE, Wilson MC, Blair A, Satchwell TJ, Meinders M (2017). An immortalized adult human erythroid line facilitates sustainable and scalable generation of functional red cells. Nat Commun.

[2] Ingley E (2012). Integrating novel signaling pathways involved in erythropoiesis. IUBMB Life.

[3] Xu T, Wang W, Zhang S, Stewart RA, Yu W (1995). Identifying tumor suppressors in genetic mosaics: the Drosophila lats gene encodes a putative protein kinase. Development.

[4] Justice RW, Zilian O, Woods DF, Noll M, Bryant PJ (1995). The Drosophila tumor suppressor gene warts encodes a homolog of human myotonic dystrophy kinase and is required for the control of cell shape and proliferation. Genes Dev.

[5] Wu S, Huang J, Dong J, Pan D (2003). Hippo encodes a Ste-20 family protein kinase that restricts cell proliferation and promotes apoptosis in conjunction with salvador and warts. Cell.

[6] Dong J, Feldmann G, Huang J, Wu S, Zhang N, Comerford SA (2007). Elucidation of a universal size-control mechanism in Drosophila and mammals. Cell.

[7] Zhao B, Wei X, Li W, Udan RS, Yang Q, Kim J (2007). Inactivation of YAP oncoprotein by the Hippo pathway is involved in cell contact inhibition and tissue growth control. Genes Dev.

[8] Pan DJ (2007). Hippo signaling in organ size control. Genes Dev.

[9] Yagi R, Chen LF, Shigesada K, Murakami Y, Ito Y (1999). A WW domain-containing Yes-associated protein (YAP) is a novel transcriptional co-activator. EMBO J.

[10] Kanai F, Marignani PA, Sarbassova D, Yagi R, Hall RA, Donowitz M (2000). TAZ: a novel transcriptional co-activator regulated by interactions with 14-3-3 and PDZ domain proteins. EMBO J.

[11] Plouffe SW, Lin KC, Moore JL, Tan FE, Ma S, Ye Z (2018). The Hippo pathway effector proteins YAP and TAZ have both distinct and overlapping functions in the cell. J Biol Chem.

[12] LeBlanc L, Ramirez N, Kim J (2021). Context-dependent roles of YAP/TAZ in stem cell fates and cancer. Cell Mol Life Sci.

[13] Lorthongpanich C, Messerschmidt DM, Chan SW, Hong W, Knowles BB, Solter D (2013). Temporal reduction of LATS kinases in the early preimplantation embryo prevents ICM lineage differentiation. Genes Dev.

[14] Ferguson GB, Martinez-Agosto JA (2014). Yorkie and Scalloped signaling regulates Notch-dependent lineage specification during Drosophila hematopoiesis. Curr Biol.

[15] Ferguson GB, Martinez-Agosto JA (2014). Kicking it up a Notch for the best in show: Scalloped leads Yorkie into the haematopoietic arena. Fly (Austin).

[16] Lorthongpanich C, Jiamvoraphong N, Supraditaporn K, Klaihmon P, U-pratya Y, Issaragrisil S (2017). The Hippo pathway regulates human megakaryocytic differentiation. Thromb Haemost..

[17] Lorthongpanich C, Jiamvoraphong N, Klaihmon P, Lueangamornnara U, U-pratya Y, Laowtammathron C (2020). Effect of YAP/TAZ on megakaryocyte differentiation and platelet production. Biosci Rep.

[18] Hwang SM, Jin M, Shin YH, Ki Choi S, Namkoong E, Kim M (2014). Role of LPA and the Hippo pathway on apoptosis in salivary gland epithelial cells. Exp Mol Med.

[19] Bao YJ, Nakagawa K, Yang Z, Ikeda M, Withanage K, Ishigami-Yuasa M (2011). A cell-based assay to screen stimulators of the Hippo pathway reveals the inhibitory effect of dobutamine on the YAP-dependent gene transcription. J Biochem.

[20] Liu-Chittenden Y, Huang B, Shim JS, Chen Q, Lee SJ, Anders RA (2012). Genetic and pharmacological disruption of the TEAD-YAP complex suppresses the oncogenic activity of YAP. Genes Dev.

[21] Feng JT, Gou JH, Jia J, Yi T, Cui T, Li ZY (2016). Verteporfin, a suppressor of YAP-TEAD complex, presents promising antitumor properties on ovarian cancer. Oncotargets Ther.

[22] Griffiths RE, Kupzig S, Cogan N, Mankelow TJ, Betin VM, Trakarnsanga K (2012). Maturing reticulocytes internalize plasma membrane in glycophorin A-containing vesicles that fuse with autophagosomes before exocytosis. Blood.

[23] Ran FA, Hsu PD, Wright J, Agarwala V, Scott DA, Zhang F (2013). Genome engineering using the CRISPR-Cas9 system. Nat Protoc.

[24] Sanjana NE, Shalem O, Zhang F (2014). Improved vectors and genome-wide libraries for CRISPR screening. Nat Methods.

[25] Chan SW, Lim CJ, Loo LS, Chong YF, Huang C, Hong W (2009). TEADs mediate nuclear retention of TAZ to promote oncogenic transformation. J Biol Chem.

[26] Chen L, Chan SW, Zhang X, Walsh M, Lim CJ, Hong W (2010). Structural basis of YAP recognition by TEAD4 in the Hippo pathway. Genes Dev.

[27] Bak RO, Dever DP, Porteus MH (2018). CRISPR/Cas9 genome editing in human hematopoietic stem cells. Nat Protoc.

[28] Cai H, Xu Y (2013). The role of LPA and YAP signaling in long-term migration of human ovarian cancer cells. Cell Commun Signal.

[29] Jeong GO, Shin SH, Seo EJ, Kwon YW, Heo SC, Kim KH (2013). TAZ mediates lysophosphatidic acid-induced migration and proliferation of epithelial ovarian cancer cells. Cell Physiol Biochem.

[30] Yu FX, Zhao B, Panupinthu N, Jewell JL, Lian I, Wang LH (2012). Regulation of the Hippo-YAP pathway by G-protein-coupled receptor signaling. Cell.

[31] Wang C, Zhu XY, Feng WW, Yu YH, Jeong KJ, Guo W (2016). Verteporfin inhibits YAP function through up-regulating 14-3-3 sigma sequestering YAP in the cytoplasm. Am J Cancer Res.

[32] Lorthongpanich C, Laowtammathron C, Jiamvoraphong N, Srisook P, Chingsuwanrote P, Klaihmon P (2020). YAP-depleted iPSC MUSIi012-A-2 maintained all normal stem cell characteristics. Stem Cell Res.

[33] Lorthongpanich C, Jiamvoraphong N, Supakun P, Damkham N, Terbto P, Waeteekul S (2019). Generation of a WWTR1 mutation induced pluripotent stem cell line, MUSIi012-A-1, using CRISPR/Cas9. Stem Cell Res.

[34] Bradley TR, Metcalf D (1966). The growth of mouse bone marrow cells in vitro. Aust J Exp Biol Med Sci.

[35] Marx-Blumel L, Marx C, Weise F, Frey J, Perner B, Schlingloff G (2020). Biomimetic reconstruction of the hematopoietic stem cell niche for in vitro amplification of human hematopoietic stem cells. PLoS ONE.

[36] Johnson R, Halder G (2014). The two faces of Hippo: targeting the Hippo pathway for regenerative medicine and cancer treatment. Nat Rev Drug Discov.

[37] Jansson L, Larsson J (2012). Normal hematopoietic stem cell function in mice with enforced expression of the Hippo signaling effector YAP1. PLoS ONE.

[38] Jansson L, Larsson J (2011). Enforced expression of Yap1 does not alter hematopoietic stem cell function. Exp Hematol.

[39] Donato E, Biagioni F, Bisso A, Caganova M, Amati B, Campaner S (2018). YAP and TAZ are dispensable for physiological and malignant haematopoiesis. Leukemia.

[40] An X, Schulz VP, Li J, Wu K, Liu J, Xue F (2014). Global transcriptome analyses of human and murine terminal erythroid differentiation. Blood.

[41] Paralkar VR, Mishra T, Luan J, Yao Y, Kossenkov AV, Anderson SM (2014). Lineage and species-specific long noncoding RNAs during erythro-megakaryocytic development. Blood.

[42] Pishesha N, Thiru P, Shi J, Eng JC, Sankaran VG, Lodish HF (2014). Transcriptional divergence and conservation of human and mouse erythropoiesis. Proc Natl Acad Sci U S A.

[43] An X, Schulz VP, Mohandas N, Gallagher PG (2015). Human and murine erythropoiesis. Curr Opin Hematol.

[44] Li J, Gao E, Vite A, Yi R, Gomez L, Goossens S (2015). Alpha-catenins control cardiomyocyte proliferation by regulating Yap activity. Circ Res.

[45] Schlegelmilch K, Mohseni M, Kirak O, Pruszak J, Rodriguez JR, Zhou D (2011). Yap1 acts downstream of alpha-catenin to control epidermal proliferation. Cell.

[46] Goode DK, Obier N, Vijayabaskar MS, Lie ALM, Lilly AJ, Hannah R (2016). Dynamic gene regulatory networks drive hematopoietic specification and differentiation. Dev Cell.

[47] Lundin V, Sugden WW, Theodore LN, Sousa PM, Han A, Chou S (2020). YAP regulates hematopoietic stem cell formation in response to the biomechanical forces of blood flow. Dev Cell.

[48] Hao S, Matsui Y, Lai ZC, Paulson RF (2019). Yap1 promotes proliferation of transiently amplifying stress erythroid progenitors during erythroid regeneration. Exp Hematol.

[49] Yang CT, Ma R, Axton RA, Jackson M, Taylor AH, Fidanza A (2017). Activation of KLF1 enhances the differentiation and maturation of red blood cells from human pluripotent stem cells. Stem Cells.

[50] McDevitt MA, Shivdasani RA, Fujiwara Y, Yang H, Orkin SH (1997). A "knockdown" mutation created by cis-element gene targeting reveals the dependence of erythroid cell maturation on the level of transcription factor GATA-1. Proc Natl Acad Sci U S A.

[51] Luo H, Yu Q, Liu Y, Tang M, Liang M, Zhang D (2020). LATS kinase-mediated CTCF phosphorylation and selective loss of genomic binding. Sci Adv.

[52] Zhang JM, Smolen GA, Haber DA (2008). Negative regulation of YAP by LATS1 underscores evolutionary conservation of the Drosophila Hippo pathway. Can Res.

[53] Li Y, Wang J, Zhong W (2021). Regulation and mechanism of YAP/TAZ in the mechanical microenvironment of stem cells (review). Mol Med Rep.

[54] Rausch V, Hansen CG (2020). The Hippo pathway, YAP/TAZ, and the plasma membrane. Trends Cell Biol.

[55] Rausch V, Bostrom JR, Park J, Bravo IR, Feng Y, Hay DC (2019). The Hippo pathway regulates caveolae expression and mediates flow response via caveolae. Curr Biol.

