# Supplementary material for: YAP and TAZ play a crucial role in human erythrocyte maturation and enucleation
Source: Stem Cell Res Ther. 2022 Sep 8;13:467. doi: 10.1186/s13287-022-03166-7 (PMC9461202; doi:10.1186/s13287-022-03166-7)
Supplement: Supplementary file 1 — Additional file 1. Figure S1. Expression of pYAP, pTAZ, and their upstream mediators LATS1/2 kinases during erythroid differentiation, as analyzed by Western blot analysis. Figure S2. Effect of LPA and DH on proliferation rate of PB- and CB- CD34+ HSC at various concentration. Figure S3. Cell pellet of PB-derived erythroid cells after 10 μM LPA and DH treatment. Figure S4. Expression of YAP target genes: c-Myc, CTGF, Cyclin D1 and CYR61 after DH and LPA treatment for 11 days of PB-HSC-derived erythroblasts. Figure S5. Expression of cleaved caspase 3, a pro-apoptotic protein, after treatment of PB-CD34+ HSCs with 10 μM LPA and 10 μM DH on the terminal day of differentiation (day 18). Figure S6. Depletion of YAP/TAZ impaired erythroid differentiation from CB-CD34+ HSCs similar to PB-CD34+ HSCs. (A) Effect of LPA and DH on YAP/TAZ expression of CB-CD34+ HSC-derived erythroblasts after adding 10 μM LPA or 10 μM DH every other day for 11 days. (B) Fold increase of cells during erythroid differentiation from CB-CD34+ HSCs after treatment with LPA (green), DH (red) and control (black) (n = 3). (C) Representative cell morphology during erythroid differentiation from CB-CD34+ HSCs after LPA and DH treatment, erythroblasts (black arrow). (D) Percentage of mature erythrocytes and erythroblasts at the terminal stage of differentiation (day 20). At least 500 cells were counted in each group (n=4). Data represent the mean ± SEM. *p<0.05, Student’s t-test. Scale bar, 20 μm. [file 13287_2022_3166_MOESM1_ESM.docx]

**Additional file 1**

**Supplementary Figures**


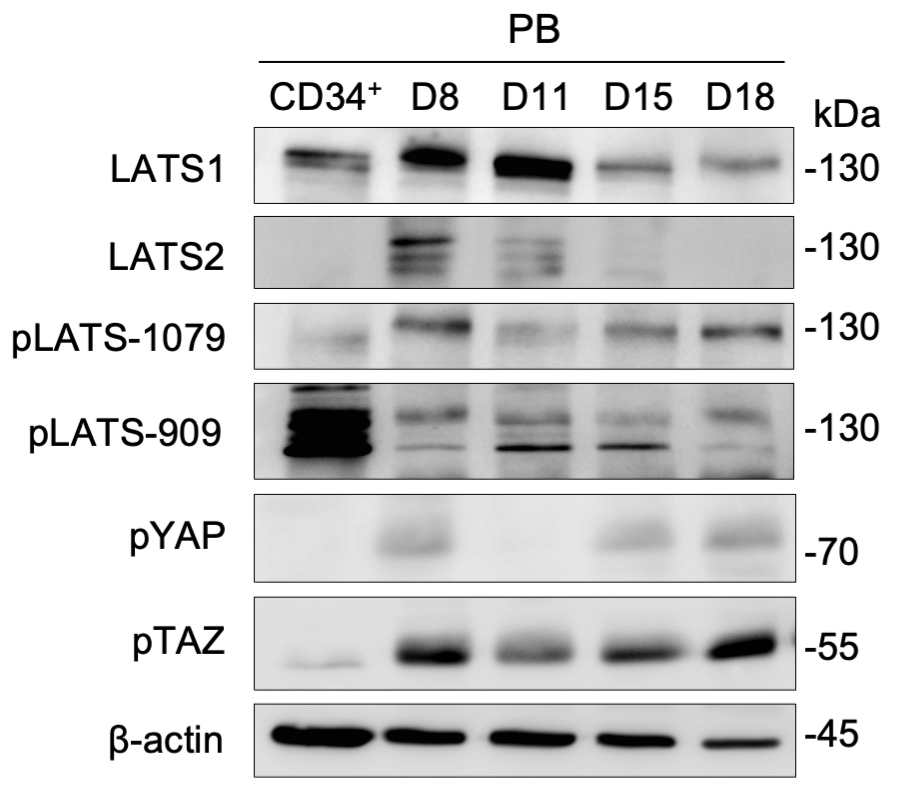


**Supplementary Figure 1.** Expression of pYAP, pTAZ, and their upstream mediators LATS1/2 kinases during erythroid differentiation, as analyzed by Western blot analysis.


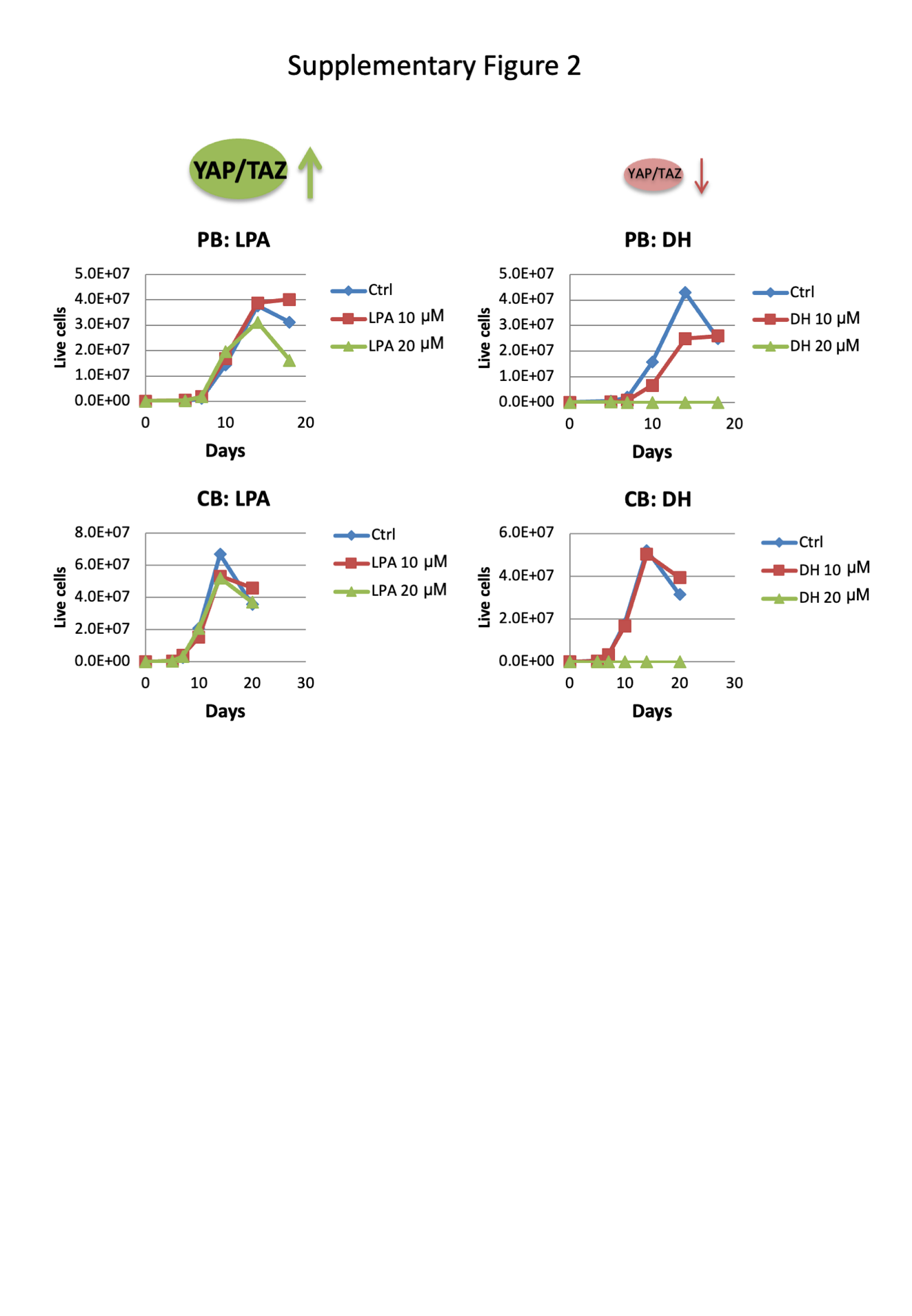


**Supplementary Figure 2.** Effect of LPA and DH on proliferation rate of PB- and CB- CD34^+^ HSC at various concentration.


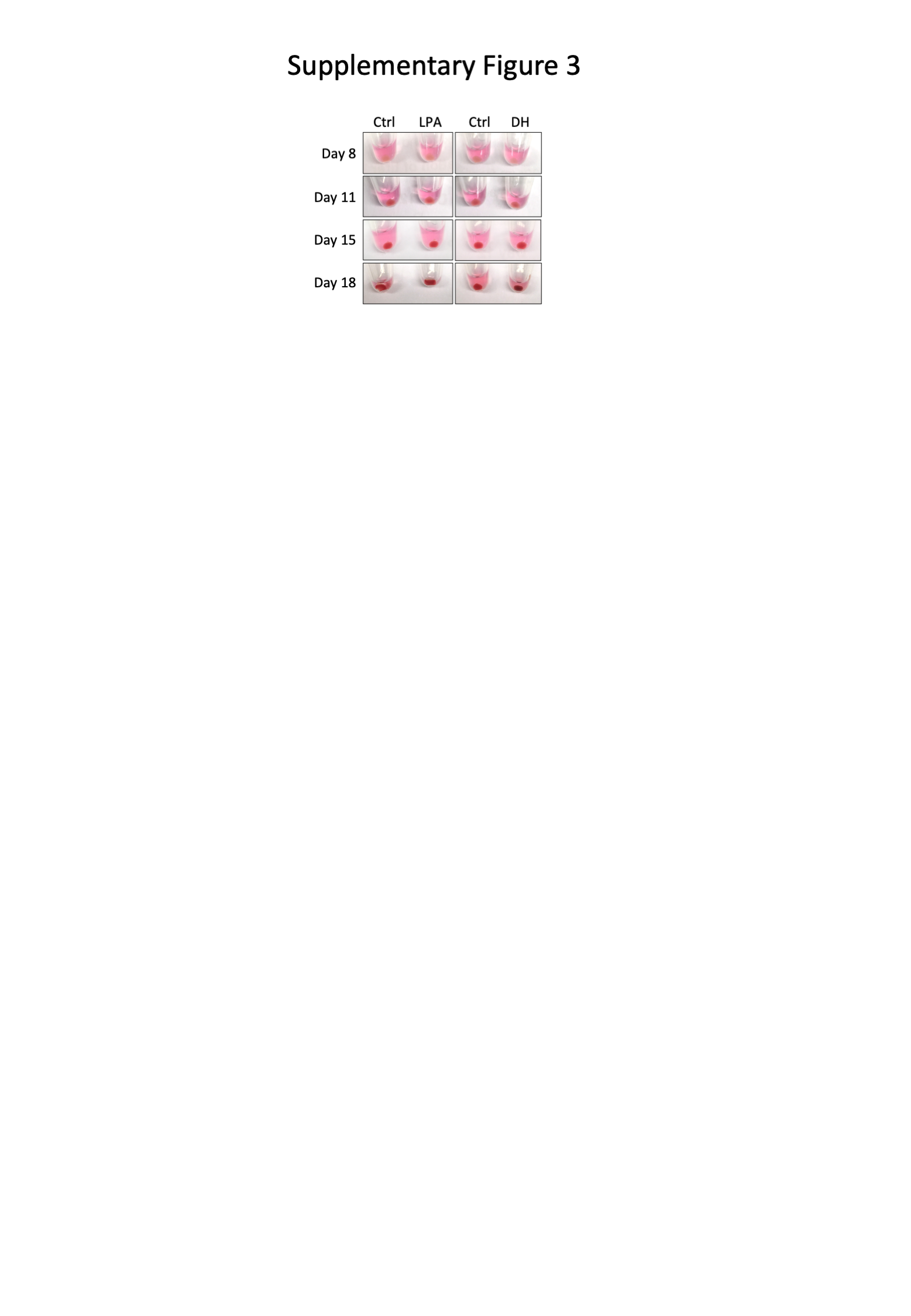


**Supplementary Figure 3.** Cell pellet of PB-derived erythroid cells after 10 μM LPA and DH treatment.

**Supplementary Figure 4.** Expression of YAP target genes: *c-Myc*, *CTGF*, *Cyclin D1* and *CYR61* after DH and LPA treatment for 11 days of PB-HSC-derived erythroblasts.

**
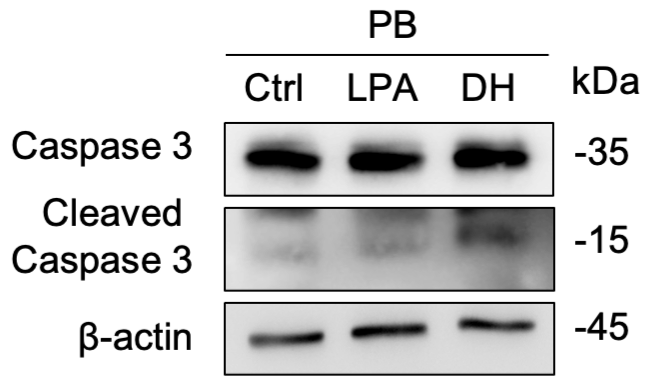
**

**Supplementary Figure 5.** Expression of cleaved caspase 3, a pro-apoptotic protein, after treatment of PB-CD34^+^ HSCs with 10 μM LPA and 10 μM DH on the terminal day of differentiation (day 18).

**
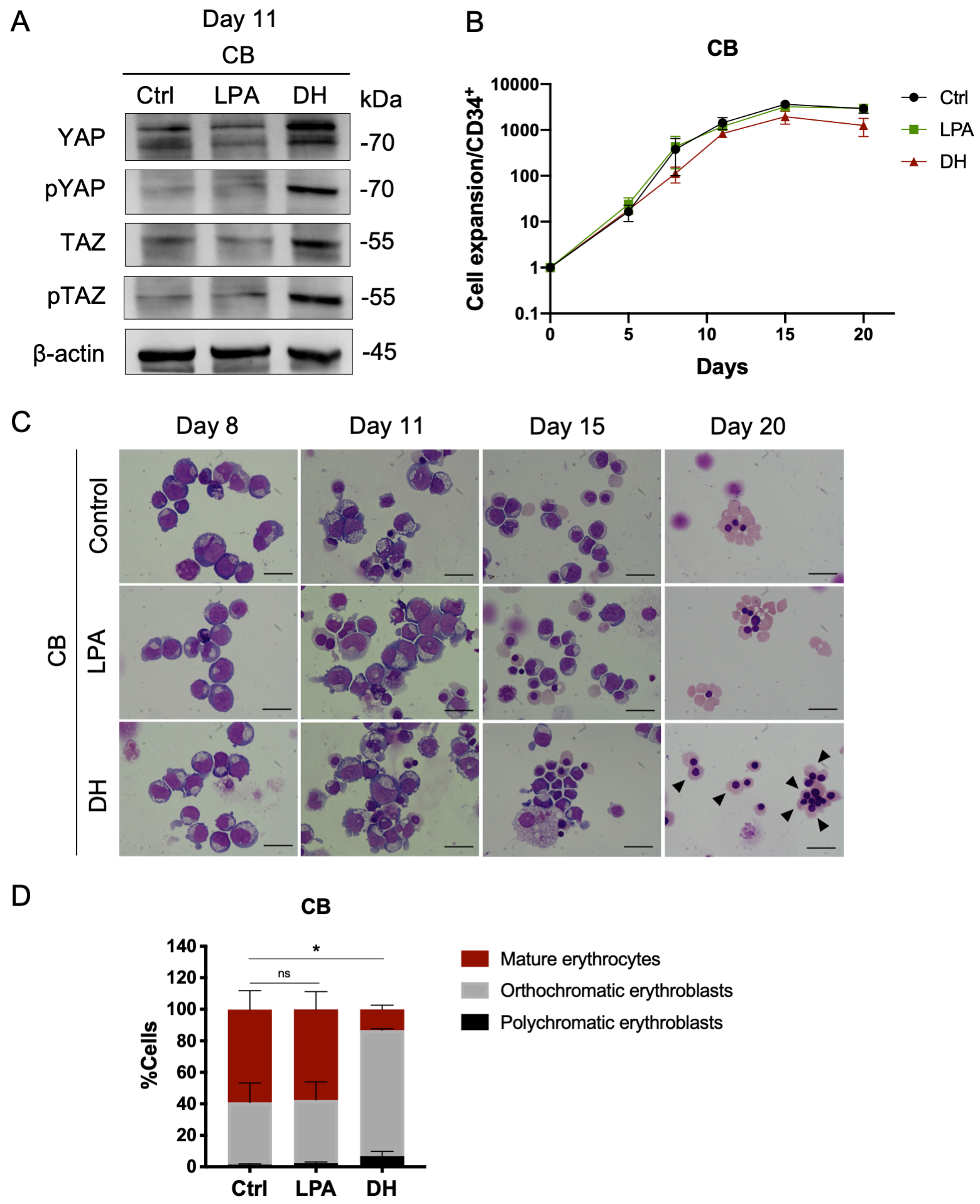
**

**Supplementary Figure 6. Depletion of YAP/TAZ impaired erythroid differentiation from CB-CD34^+^ HSCs similar to PB-CD34^+^ HSCs.** (A) Effect of LPA and DH on YAP/TAZ expression of CB-CD34^+^ HSC-derived erythroblasts after adding 10 μM LPA or 10 μM DH every other day for 11 days. (B) Fold increase of cells during erythroid differentiation from CB-CD34^+^ HSCs after treatment with LPA (green), DH (red) and control (black) (n=3). (C) Representative cell morphology during erythroid differentiation from CB-CD34^+^ HSCs after LPA and DH treatment, erythroblasts (black arrow). (D) Percentage of mature erythrocytes and erythroblasts at the terminal stage of differentiation (day 20). At least 500 cells were counted in each group (n=4). Data represent the mean ± SEM. **p*<0.05, Student’s *t*-test. Scale bar, 20 μm.

**
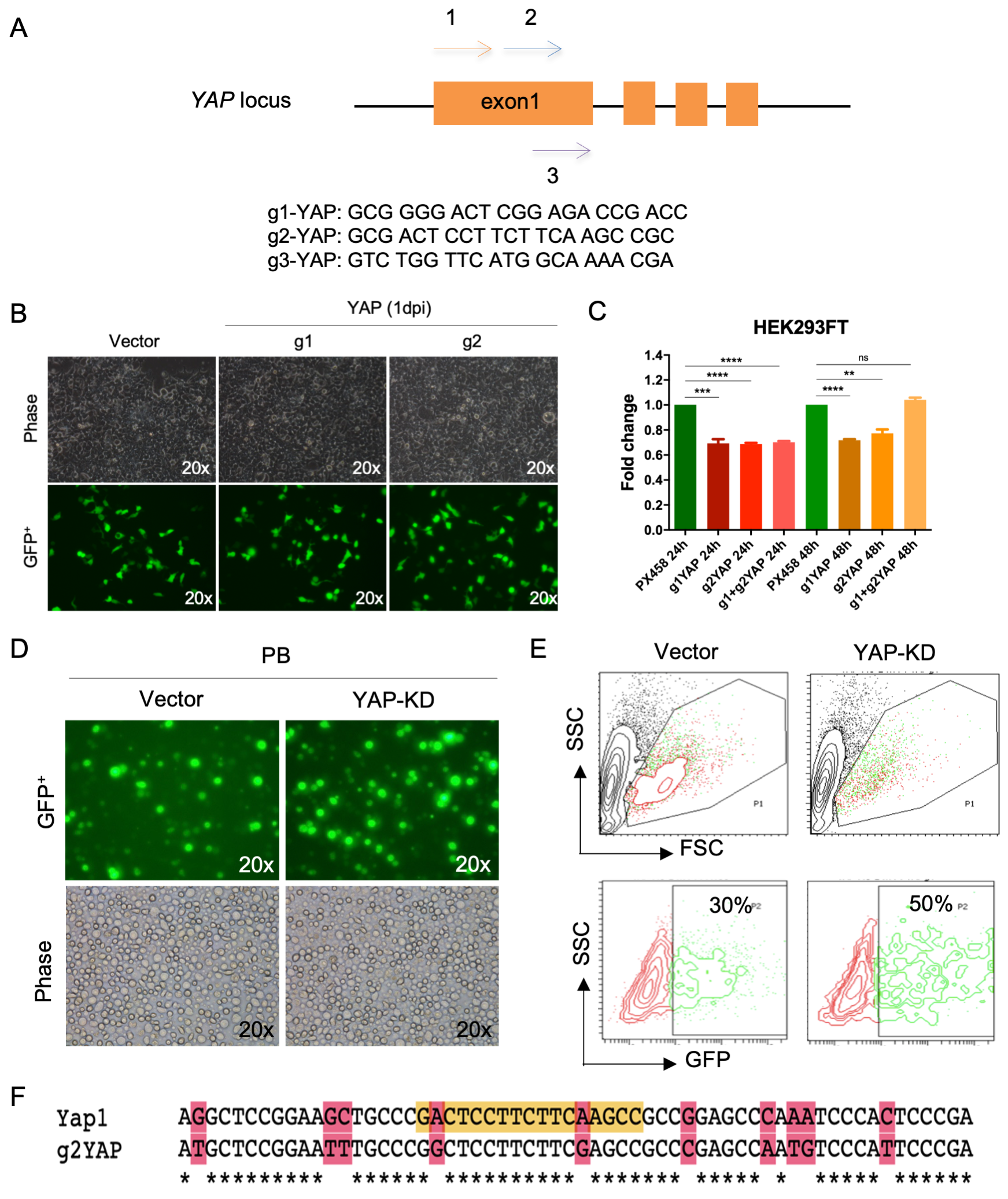
**

**Supplementary Figure 7. Sequences of gRNA and transfection efficiency of PX458-gYAP plasmid in HSC derived pro-erythroblasts.** (A) Sequences of gRNA targeting YAP at exon 1. (B) GFP^+^ HEK293FT cells containing CRISPR/Cas9 plasmid at 1 day post infection (dpi). (C) Fold change of *YAP* mRNA expression in HEK293FT cells after transfection without antibiotic selection. Data represent 2 independent experiments. (D) GFP^+^ pro-erythroblasts derived from PB-CD34^+^ HSCs containing PX458-GFP (vector) and PX458-GFP_gYAP at 24-hours post-nucleofection. (E) Percentage of GFP^+^ cells analyzed by flow cytometry at 24-hours post-nucleofection. (F) Sequences of pooled erythroblast cell transfected with PX458-GFP-gYAP (g2YAP) aligned to *YAP* genomic sequence from GenBank data base. Targeted of sgRNA sequence was highlight in yellow. Indel mutation were highlight in pink. Magnification, 20x.

**Supplementary Figure 8.** Expression of YAP target genes: *c-Myc*, *Cyclin D1* and *CYR61* after YAP-KD of PB-HSC at 3 dpi.

**Supplementary Figure 9.** Expression of YAP target gene: *c-Myc* after YAP overexpression after 6 dpi of CB-HSC-derived erythroblasts.


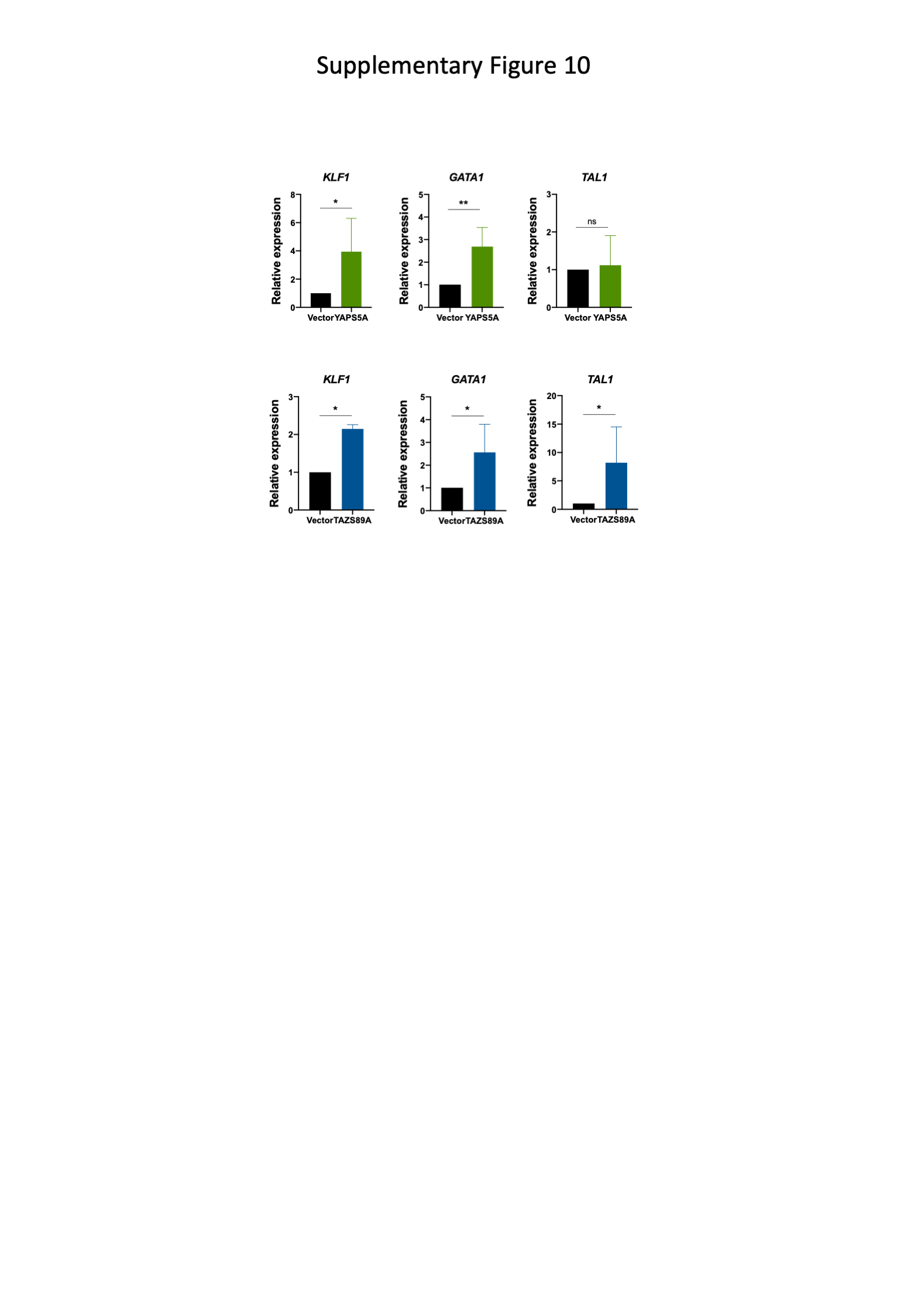


**Supplementary Figure 10.** Erythroid-specific gene expression after YAP and TAZ overexpression analyzed at 3 dpi.


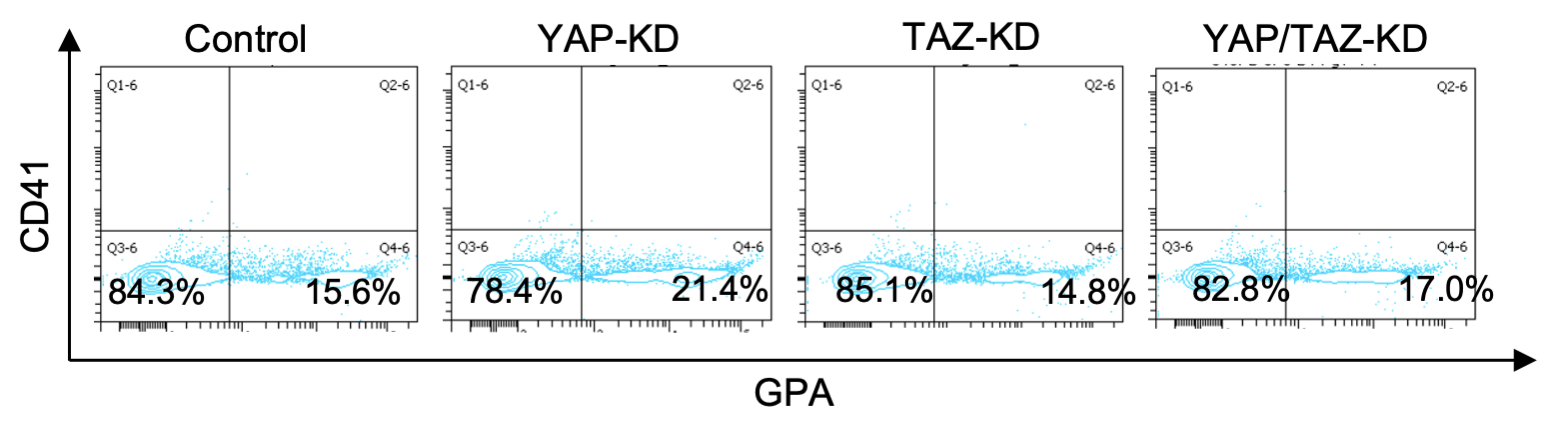


**Supplementary Figure 11.** **Flow cytometry scatters plot of HSC-derived GEMM, BFU-E, and GM in methylcellulose for 14 days.** Cells were stained with Glycophorin A (GPA), an erythroid-specific marker, and CD41, a megakaryocyte specific marker of YAP-KD, TAZ-KD, and YAP/TAZ-KD. Double negative of CD41 and GPA (CD41^-^GPA^-^) was determined to granulocyte/monocyte population. CFU-GEMM: colony-forming unit granulocyte/erythroid/macrophage/megakaryocyte, CFU-GM: colony-forming unit granulocyte/macrophage, and BFU-E: Burst-forming unit erythroid.

**Supplementary Tables**

**Supplementary Table 1.** Guide RNA (gRNA) sequences

| **Primer name** | **Sequence 5’🡪3’** | **PAM sequence** |
| --- | --- | --- |
| YAP_gRNA1_Top | CACCGCGGGGACTCGGAGACCGACC | TGG |
| YAP_gRNA1_Bottom | AAACGGTCGGTCTCCGAGTCCCCGC |  |
| YAP_gRNA2_Top | CACCGCGACTCCTTCTTCAAGCCGC | CGG |
| YAP_gRNA2_Bottom | AAACGCGGCTTGAAGAAGGAGTCGC |  |
| YAP_gRNA3_Top | CACCGTCTGGTTCATGGCAAAACGA | GGG |
| YAP_gRNA3_Bottom | AAACTCGTTTTGCCATGAACCAGAC |  |

*Underline sequences indicate *BbsI* restriction site for cloning

**Supplementary Table 2.** Primer sequences for quantitative real-time reverse transcription polymerase chain reaction (qRT-PCR)

| **Genes** | **Direction** | **Sequence (5'🡪3')** | **Probe number** |
| --- | --- | --- | --- |
| *GAPDH* | F | AGCCACATCGCTCAGACAC | 60 |
|  | R | GCCCAATACGACCAAATCC |  |
| *GATA1* | F | CACTGAGCTTGCCACATCC | 26 |
|  | R | ATGGAGCCTCTGGGGATTA |  |
| *KLF1* | F | TTACGGAAAATCCGACAAGC | 5 |
|  | R | TGCACGACAGTTTGGACATC |  |
| *TAL1* | F | CCCTTGTCTCCCGTTAACAC | 85 |
|  | R | GGGCAAGGTAGAATCCATGA |  |
| *TAZ* | F | GTATCCCAGCCAAATCTCGT | 84 |
|  | R | TTCTGCTGGCTCAGGGTACT |  |
| *YAP* | F | GATCTTCCTTTACCCCTCAACTTT | 61 |
|  | R | CACAGTACTGCTACAACACTGAGGT |  |
